# Efficient regeneration system for rapid multiplication of clean planting material of *Ensete ventricosum* (Welw.) Cheesman

**DOI:** 10.1007/s11627-017-9867-9

**Published:** 2017-12-01

**Authors:** Jaindra Tripathi, Jonathan Matheka, Ibsa Merga, Endale Gebre, Leena Tripathi

**Affiliations:** 1 International Institute of Tropical Agriculture (IITA), Nairobi, Kenya; 2 Ethiopian Institute of Agriculture Research (EIAR), Addis Ababa, Ethiopia

**Keywords:** *Ensete ventricosum*, Bedadeti, Corm discs, Intercalary meristem, Micropropagation

## Abstract

Enset (*Ensete ventricosum* (Welw.) Cheesman) is an economically important staple food crop in Ethiopia, especially in the southern and southwestern regions. It is called “false banana” due to its resemblance to banana, but inability to produce any edible fruit. The crop is clonally propagated using field-grown suckers. This study reports the development of a robust regeneration technique to propagate large numbers of plantlets using corm discs containing intercalary meristematic tissues. Hundreds of shoot buds were induced from corm discs of enset cultivar ‘Bedadeti’ cultured on Murashige and Skoog (MS) medium supplemented with 1.5 mg L^−1^ 2,4-dichlorophenoxyacetic acid, 0.216 mg L^−1^ zeatin, and 2 g L^−1^ activated charcoal. The shoot buds were regenerated into complete plantlets when transferred onto MS medium supplemented with 1 mg L^−1^ 6-benzylaminopurine and 2 g L^−1^ activated charcoal. More than 100 plantlets were generated in 4 mo from corm discs isolated from a single *in vitro* mother plantlet. Well-rooted plantlets were acclimatized in soil with 100% success, and did not show any apparent phenotypic abnormalities under glasshouse conditions. This efficient regeneration system could be very useful for the rapid multiplication of clean pathogen-free planting material.

*Ensete ventricosum* (Welw.) Cheesman (enset) is a large, perennial, monocotyledonous herbaceous plant that belongs to the family Musaceae, and is also known as “false banana.” The plant appears similar to banana but takes several years to flower, and its small-sized banana-like fruits are seeded and inedible. It is a staple food crop grown on over 321,362 ha in the southern part of Ethiopia (Central Statistics Authority 2013), where it provides food security for more than 20 million people (Yemataw *et al.* 2014). The enset plant is grown as an intercrop with coffee, maize, sorghum, and millet, and produces large quantities of starch in its underground rhizome or corm, and the pseudostem that can reach a height of 5–12 m. Due to its big corm and thick pseudostem, the crop can survive under drought conditions with little adverse effects for several years without rain. This makes it a food security crop during famine and drought conditions. Enset provides a greater amount of food per unit area than most cereals. According to the Country Information Brief (FAOSTAT 1995), about 40 to 60 plants covering 250–375 m^2^ of land can provide enough food for a family of five to six people for a year. It is also a source of animal feed and a tough fiber (Bezuneh *et al.* 1967).

The sustainability of enset agriculture is threatened by various factors including increased population pressure, which is associated with more extreme cultivation, degradation of the soil, and changing environmental temperatures (Quimio and Tessera 1996). Enset cultivation is also threatened by various diseases caused by fungi, bacteria, and viruses. The bacterial wilt disease, caused by *Xanthomonas campestris* pv. *musacearum* (Xcm), is the deadliest disease affecting production of enset (Yirgou and Bradbury 1968).

Enset plants produce flowers in 5–7 y, and farmers typically harvest the plants before flowering. Propagation of enset through seeds is difficult due to the low germination rate (12%), and long germination time (12 wk) (Karlsson *et al.* 2013). Wild enset plants are produced from seeds, whereas cultivated enset is primarily vegetatively propagated through suckers produced from corms of mother plants. However, enset, unlike banana, does not produce suckers, as it possesses a single apical dominant bud, which prevents lateral bud development. The farmers’ traditional technique that leads to multiple sucker development is to isolate the corm from the field-grown plant and bury it in the soil after removing the apical tip. However, this process is very laborious and time-consuming and results in a poor propagation rate and diseased planting materials. An efficient clonal propagation system that yields pathogen-free planting material is highly desirable.

Micropropagation of enset has been reported (Afza *et al.* 1996; Negash *et al.* 2000; Chimsa 2003; Birmeta and Welander 2004; Konobo 2014), but requires further improvement. Most studies have focused on the regeneration of plantlets from proliferated axillary buds. However, high exudation of phenolic compounds and endogenous bacteria were reported as the major challenges to successful micropropagation. Therefore, a strategy to overcome these constraints needs to be devised.

The present study was undertaken to develop an efficient micropropagation system for enset cultivar ‘Bedadeti’ using corm discs containing intercalary meristems. ‘Bedadeti’ is one of the most widely distributed enset cultivars with a good yield of fiber and tolerance to bacterial wilt disease and drought, and is traditionally used for treating some human diseases (Yemataw *et al.* 2016).

*In vitro* plantlets of enset cultivar ‘Bedadeti’ were obtained from the Ethiopian Institute of Agriculture Research (EIAR), Addis Ababa, Ethiopia. The plantlets were acclimatized and established in the screen house for maintenance. The shoot tips isolated from screen house-grown plants were used as sources of explants for initiation and multiplication of *in vitro* plantlets.

The shoot tip explants (diameter 2.5–3.0 cm; length 4–5 cm) were isolated from 4-mo-old screen house-grown plants. Extra sheaths were removed, and the explants were soaked in 70% (*v/v*) ethanol for 5, 10, 15, 20, or 30 min. This was followed by soaking the explants in 2.5% (*v/v*) NaOCl (JIK Bleach containing 3.85% (*w/v*) NaOCl, Orbit Chemical Industries Limited, Nairobi, Kenya) with 0.02% (*v/v*) Tween®-20 (Duchefa Biochemie, Haarlem, Netherlands) for 20 min. The explants were rinsed three times with sterile distilled water and soaked again in 2.5% (*v/v*) NaOCl with 0.02% (*v/v*) Tween®-20 for 10 min, then rinsed again three times with sterile distilled water and blotted dry using sterile tissue papers. The outer leaf sheath was peeled off, and the explants were reduced to a length of about 2.0 to 2.5 cm before being split into halves longitudinally. Then the explants were cultured on regeneration medium (RM1, Table 1) in a dark room at 26 ± 2°C, and monitored daily for any fungal and/or bacterial contamination. The percentage of clean explants was calculated as the number of explants devoid of fungal and bacterial contamination, divided by the total number of explants used for surface sterilization × 100. To regenerate multiple shoots, the explants were transferred to proliferation medium (PM1, Table 1), and kept in the dark at 26 ± 2°C for 2 wk. The cultures were transferred to a 16-h photoperiod with a photon flux density of 50 μmol m^−2^ s^−1^ provided by white fluorescent tube light (Osram, Nairobi, Kenya) and sub-cultured onto fresh PM1 every 2 wk. The shoots initiated on PM1 were isolated and transferred to fresh PM1 for shoot elongation and rooting.

**Table 1.** Composition of media^z^ used for regeneration of *Ensete ventricosum* (Welw.) Cheesman (enset)

Name of medium	Ascorbic acid (10 mg L^−1^)	BAP (mg L^−1^)	2,4-D (mg L^−1^)	Zeatin (mg L^−1^)	Activated charcoal (2 g L^−1^)
RM1	Yes	1.0	0.0	0.0	No
PM1	Yes	2.5	0.0	0.0	Yes
PM2	Yes	1.0	0.0	0.0	Yes
PM3	Yes	5.0	0.0	0.0	Yes
PM4	Yes	1.0	0.0	0.0	No
PM5	Yes	2.5	0.0	0.0	No
PM6	Yes	5.0	0.0	0.0	No
CIM1	Yes	0.0	1.5	0.216	Yes
CIM2	Yes	0.0	1.5	0.216	No
MBM1	Yes	0.0	0.0	0.0	Yes
MBM2	Yes	0.0	0.0	0.0	No

All chemicals purchased from Duchefa Biochemie, Haarlem, Netherlands.

*RM* regeneration medium, *PM* proliferation medium, *CIM* callus induction medium, *MBM* MS basal medium, *2,4-D* 2,4-dichlorophenoxyacetic acid, *BAP* 6-benzylaminopurine

^z^In addition to components shown in the table, all media contain Murashige and Skoog (1962) salts and vitamins, 30 g L^−1^ sucrose, and 2 g L^−1^ Gelrite™, and are adjusted to pH 5.8 prior to autoclaving.

The effect of 2 g L^−1^ activated charcoal (Duchefa Biochemie) was also evaluated by sub-culturing the shoot tips on PM1, with or without activated charcoal (PM5, Table 1).

Fully developed *in vitro* plantlets, 5–6 cm tall with a thick pseudostem, were selected for isolation of corm explants. Fine transverse sections of corm (0.2–0.3 mm thick) containing intercalary meristematic tissues were cut after removal of the roots, leaves, and pseudostem (Fig. 1). Three thin corm discs were excised from each *in vitro* plantlet.

**Figure 1.**
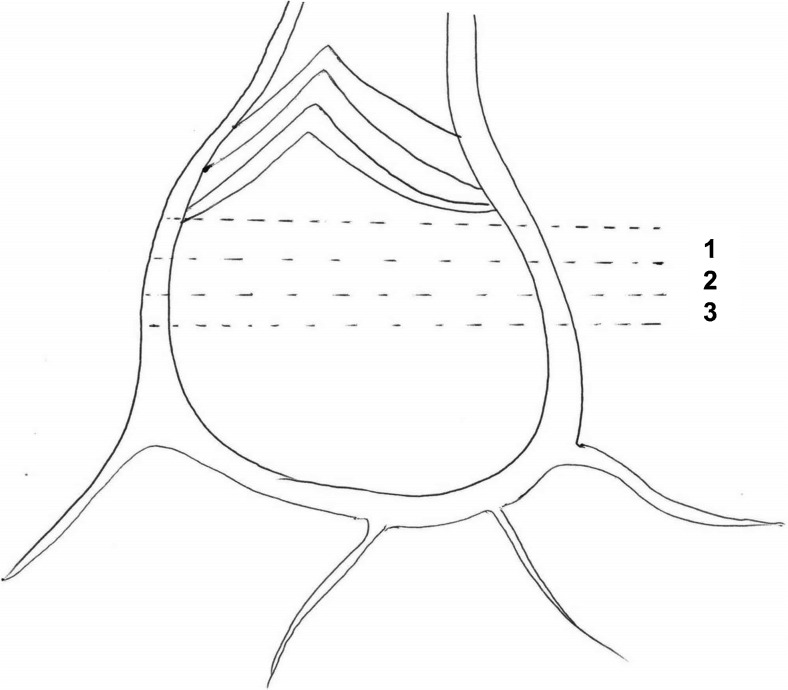
Diagrammatic picture of corm of *Ensete ventricosum* (Welw.) Cheesman (enset) showing positions for cutting discs as explants.

The corm discs containing intercalary meristems were used as explants for callus induction. Six explants were cultured in each 95-mm Petri dish containing callus induction medium either with 2 g L^−1^ activated charcoal (CIM1, Table 1) or without activated charcoal (CIM2, Table 1). Cultures were incubated in a dark room at 26 ± 2°C for 4 wk. The explants were transferred to fresh CIM1 or CIM2 medium for an additional 4 wk.

After 8 wk, the explants with multiple buds were divided into four pieces and transferred to proliferation medium with activated charcoal (PM2, Table 1) for shoot regeneration. The cultures were incubated for 4 wk at 26 ± 2°C in a 16-h photoperiod. The regenerated shoots were separated from the explants, and individual shoots were transferred to fresh PM2 medium for elongation and rooting. The number of regenerated shoots per corm disc and the total number of complete plantlets regenerated from three corm discs isolated from a single *in vitro* mother plantlet were recorded. The experiment was performed three times with 60 explants tested for each treatment.

The ability of corm discs containing intercalary meristematic tissues to induce multiple shoot buds was also evaluated using different types of proliferation media. Corm discs were cultured on MS (Murashige and Skoog 1962) basal medium (MBM1-MBM2, Table 1), or MS medium containing 1, 2.5, or 5 mg L^−1^ 6-benzylaminopurine (BAP, Duchefa Biochemie) and supplemented with 2 g L^−1^ activated charcoal (PM1–3, Table 1), or without activated charcoal (PM4–PM6, Table 1). Six explants were cultured in each 95-mm Petri dish at 26 ± 2°C in a dark room for 4 wk. The ability of explants to induce multiple buds was recorded after 4 wk of culture. The explants with induced multiple buds were transferred to the same fresh medium for an additional 4 wk.

Proliferation media supplemented with 1.0, 2.5, or 5.0 mg L^−1^ BAP, with or without activated charcoal (PM1–PM6, Table 1), were tested to establish the efficacy of each medium to regenerate fully developed shoots. The explants containing multiple buds on the upper surface were divided into four pieces, and cultured on proliferation medium PM1–PM6 in a 16-h photoperiod provided by white fluorescent tube light at 26 ± 2°C for 4 wk. The regenerated shoots were transferred to fresh medium for shoot elongation and rooting. The total number of shoots regenerated from three corm discs isolated from a single *in vitro* mother plantlet was recorded. Sixty explants were tested for each treatment, and each set of experiments was performed three times.

Well-rooted plantlets were weaned for hardening in small disposable plastic cups (diameter 10 cm) containing sterile forest soil under a transparent polythene tent for 4 wk in a screen house under natural conditions. These plants were gradually transferred to big plastic pots (diameter 30 cm) for proper growth in the screen house. Plants were observed up to 6 mo for any phenotypical abnormalities.

Data were collected from three independent experiments and analyzed using the Minitab 2017 program (www.minitab.com). One-way analysis of variance (ANOVA) was performed on data for the number of clean explants after surface sterilization, the number of explants with multiple buds, the number of shoots regenerated from each corm disc, and the total number of complete plantlets for each treatment. Means and standard deviations were calculated.

All pictures were taken using a Nikon D7100 camera. Microscopic pictures were taken using a Nikon Stereo microscope SMZ 1500 equipped with a Nikon digital color camera system DS-Fi1-U2 (Nikon Corporation, Tokyo, Japan), consisting of the NIS-Elements software package attached to a Dell computer.

Initial attempts to initiate *in vitro* cultures from shoot tip explants derived from screen house-grown plants failed due to endophytic bacteria secreted by the explants, which appeared as a milky white or yellowish smear on the medium. Scientists have also reported total loss of *in vitro* cultures due to endophytic bacterial contamination (Afza *et al.* 1996; Birmeta and Welander 2004). In addition to hidden endophytic bacteria, many explants were lost due to other microbial contaminants. Consequently, the surface sterilization step of the protocol was modified to reduce the contamination.

In this study, the surface sterilization technique was improved by increasing the duration of soaking the shoot tip explants in 70% (*v/v*) ethanol from 5 to 30 min. The highest frequency of clean explants (83.3%) was obtained when explants were soaked in 70% (*v/v*) ethanol for 30 min (Fig. 2). Some explants were observed to be free from contamination for short periods (7–14 d) before endophytic bacteria appeared in the culture medium around the explant. In most surface sterilization protocols for enset, the explants were soaked in 70% (*v/v*) ethanol for between 0.5 and 5 min (Afza *et al.* 1996; Negash *et al.* 2000; Birmeta and Welander 2004; Konobo 2014). An additional modification included a double sterilization step adapted from banana tissue culture that involved sequential soaking of the explants in 2.5% (*v/v*) NaOCl for 20 min and then for 10 min. The explants surface sterilized using the modified protocol remained free of microbial contaminants (both surface and endogenous) in over 80% of the cultures initiated from screen house-grown plants.

**Figure 2.**
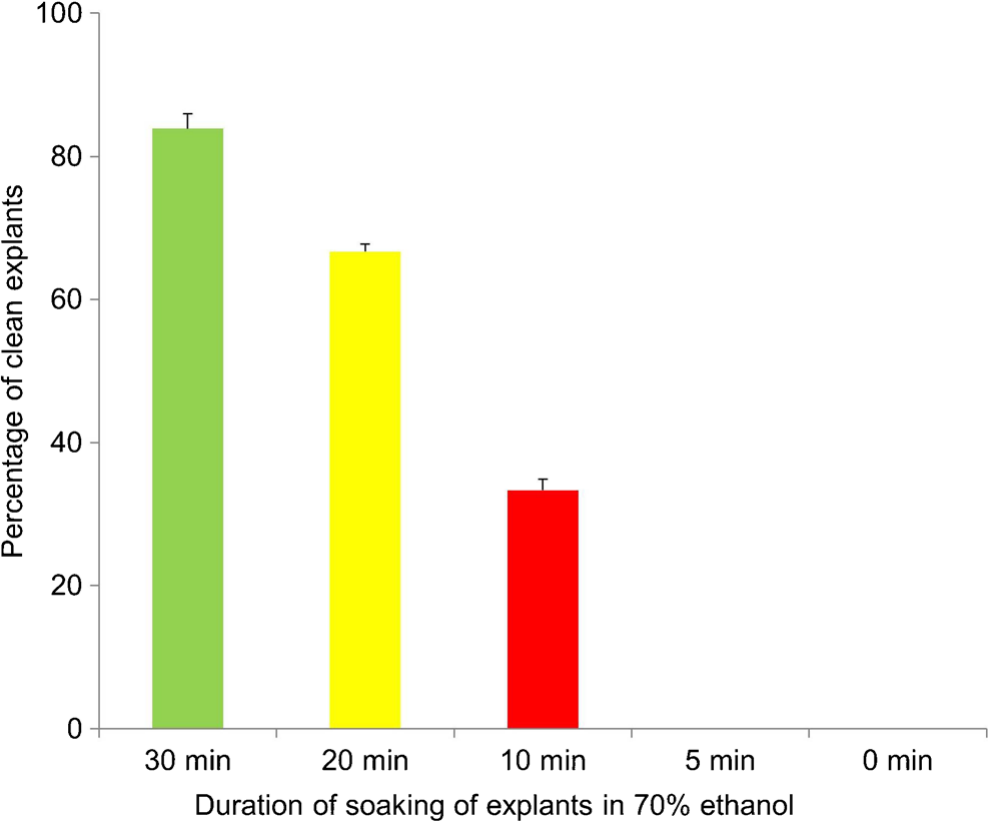
Effect of duration of soaking of shoot tip explants in 70% ethanol to obtain clean explants for regeneration of *Ensete ventricosum* (Welw.) Cheesman (enset). All values are presented as mean ± Std.

Shoot tips isolated from *in vitro* plantlets were sub-cultured on proliferation medium (PM1), with or without (PM5) activated charcoal for multiplication. The explants produced well-developed shoots with many shoot buds on the medium containing activated charcoal in 4 wk (data not included). The shoot buds were isolated and then cultured on PM1 medium for multiplication and maintenance of the plantlets. The explants transferred to proliferation medium (PM5) without activated charcoal did not perform well. The medium and the explants turned black due to phenolic compounds secreted from the cut surfaces. Phenolic compounds were the biggest problem in enset micropropagation (Negash *et al.* 2000; Birmeta and Welander 2004). This study clearly demonstrated that the addition of activated charcoal in medium improved regeneration of shoots.

The corm discs were cultured on CIM1 and CIM2 to develop callus. However, no callus was observed on explants cultured on either medium. The corm disc containing intercalary meristematic tissues cultured on CIM1 with activated charcoal became a convex dome-like structure within the first 2 wk of culture. Gradually, after 4 wk post culture, tiny creamy-white multiple buds developed, which covered the entire upper surface of the corm slices. About 86–90% of explants cultured on CIM1 showed development of multiple buds (Table 2). All three corm slices cut from each *in vitro* plantlet developed multiple buds. Different stages in the development of multiple buds are shown in Fig. 3 *a–d*. The explants with induced multiple buds were transferred to fresh CIM1 medium for an additional 4 wk. Those explants cultured on CIM2 medium without activated charcoal turned black and no buds were observed.

**Table 2.** Regeneration of *Ensete ventricosum* (Welw.) Cheesman (enset) using corm discs containing intercalary meristems

SN	Callus induction medium^z^	Efficiency of bud induction at 4 wk (%)^ya^	Shoot regeneration medium	Number of shoots initiated per corm disc^a^	Number of complete plants per corm disc^a^	Multiplication rate^xa^
1	CIM1	88.7 ± 2.3	PM2	35.5 ± 0.7	35.5 ± 0.7	106.6 ± 2.2
2	CIM2	0 ± 0	–	0 ± 0	0.0	0.0

^a^Values are mean ± Std.

^z^Explants produced multiple buds instead of callus.

^y^Efficiency of bud induction = number of explants produced multiple buds / total number of explants cultured × 100.

^x^Multiplication rate = total number of plantlets from three corm discs isolated from one *in vitro* mother plantlet.

**Figure 3.**
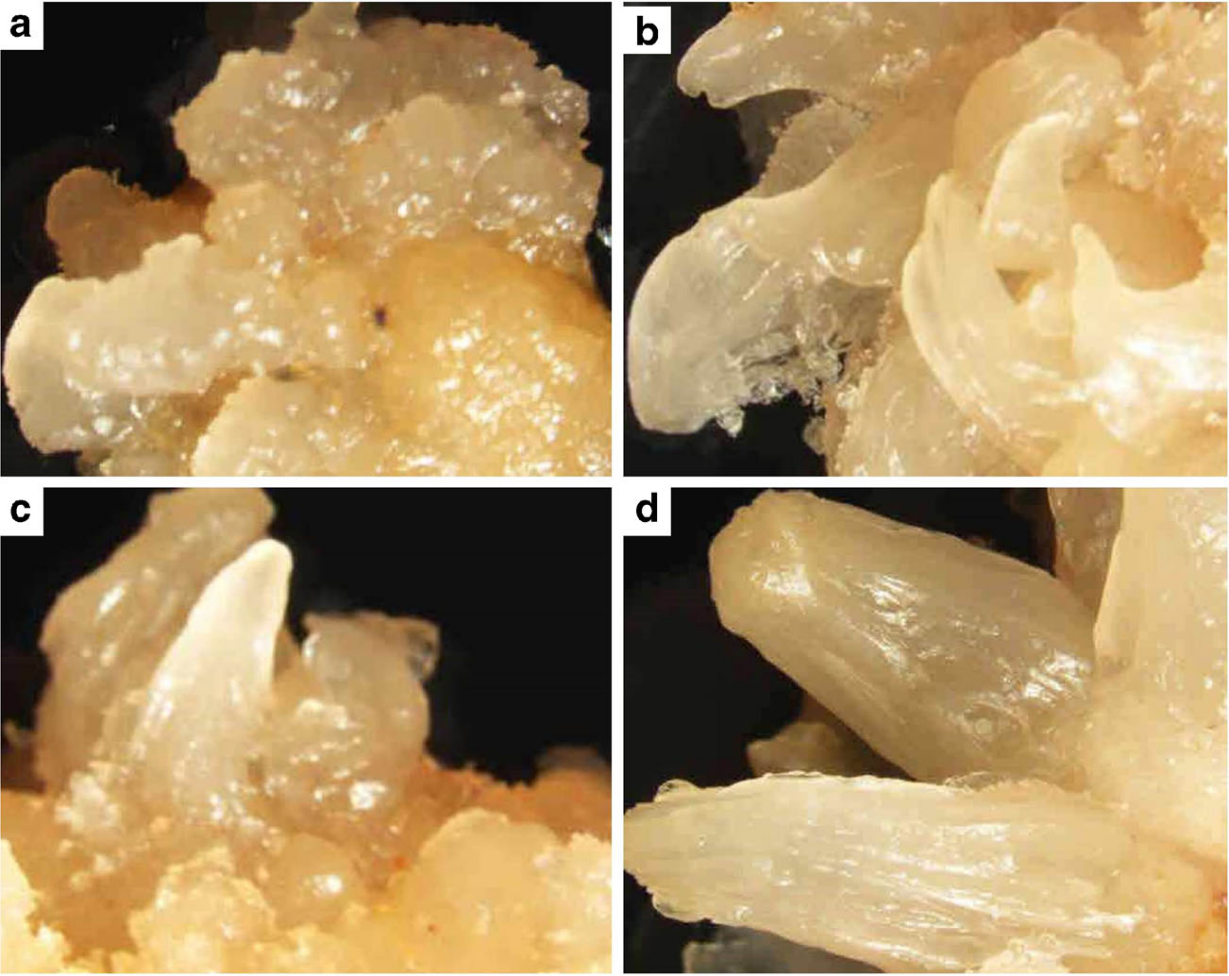
Different stages of multiple buds induced from corm discs of *Ensete ventricosum* (Welw.) Cheesman (enset) cultured on callus induction medium (CIM1). (*a*) Multiple buds from corm disc at 2 wk post culture, (*b*, *c*) multiple buds at 3 wk post culture, (*d*) multiple buds at 4 wk post culture. All photographs were taken by SMZ stereomicroscope connected with Nikon camera and desktop computer.

After 8 wk, the explants with clusters of tiny multiple buds were transferred to shoot proliferation medium (PM2) containing 1 mg L^−1^ BAP and activated charcoal. The multiple buds turned green and developed shoots in 4 wk. These regenerated shoots were separated and transferred to the same medium for an additional 4 wk for shoot elongation and development of roots. Different stages of regeneration are shown in Fig. 4 *a–f*. Each corm disc produced about 36 complete plantlets in 4 mo. A total of about 106 well-developed plantlets were obtained from three corm sections isolated from each *in vitro* mother plantlet (Table 2). The development of multiple buds from corm discs containing intercalary meristematic tissues is similar to the development of sucker buds in the traditional method, where farmers remove the apical dominance and bury the corm in soil for sucker production. In this study, apical dominance was removed by cutting off the apex and isolating the corm section from the lower section. The intercalary meristems in the axil of the leaf sheath were activated for bud initiation.

**Figure 4.**
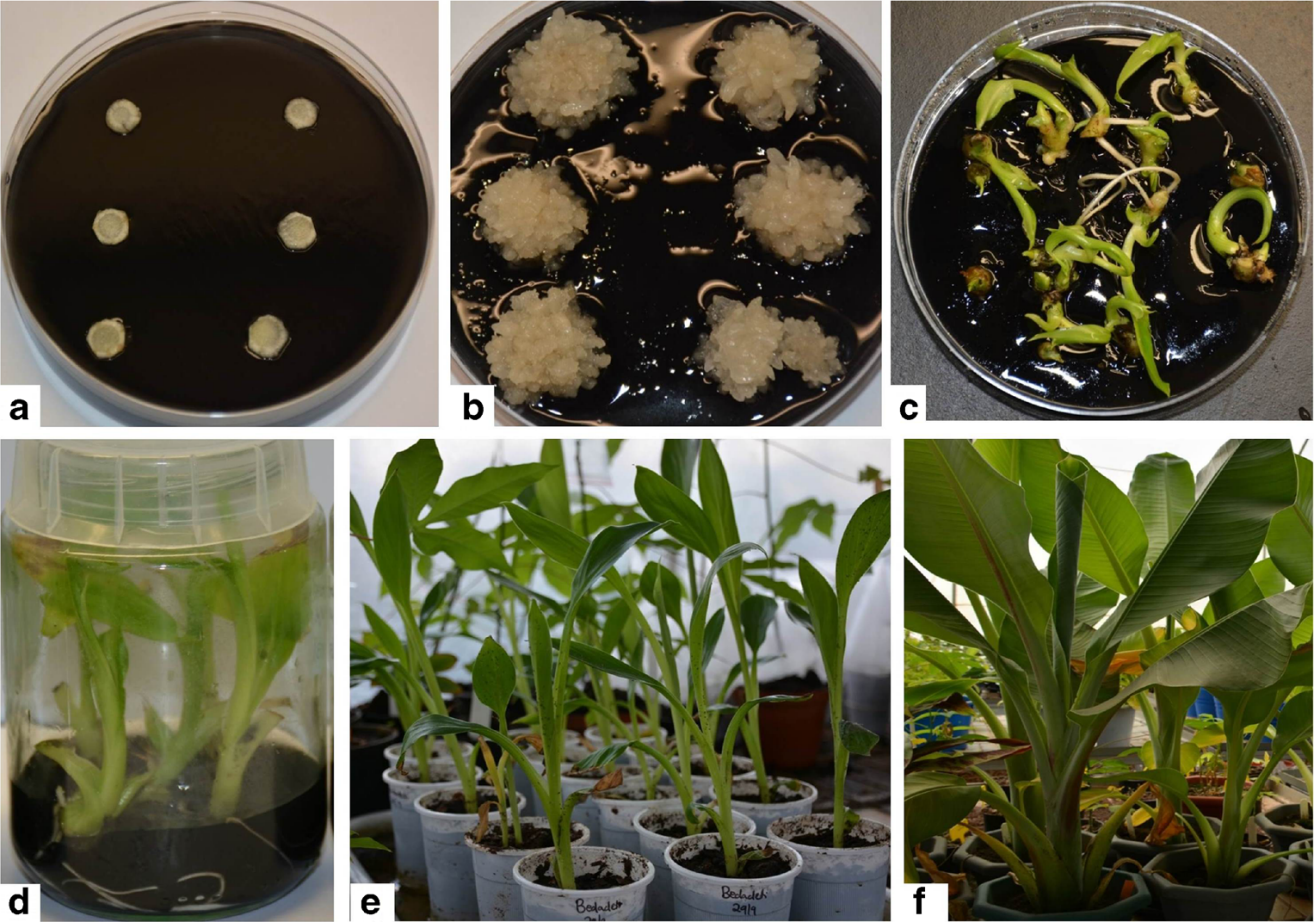
Regeneration of complete plants of *Ensete ventricosum* (Welw.) Cheesman (enset) using corm discs isolated from *in vitro* plantlets. (*a*) Corm discs cultured on callus induction medium (CIM1), (*b*) multiple buds on upper surface of corm discs cultured on CIM1, (*c*) regeneration of shoot buds upon transferring to proliferation medium (PM2), (*d*) well-rooted plantlets on PM2, (*e*) plants weaned in soil in plastic cups, (*f*) fully grown plants in pots.

The corm discs cultured on MS medium containing 0.0, 1.0, 2.5, and 5.0 mg L^−1^ BAP supplemented with activated charcoal showed induction of multiple buds. The efficiency of bud induction (*i.e.*, percentage of corm discs showing the induction of multiple buds) varied with the concentration of BAP in the proliferation medium (Table 3). The response for bud induction was slightly higher on corm discs cultured on proliferation medium containing 1.0 mg L^−1^ BAP (PM2, 81.7 ± 14.1%) compared to 2.5 mg L^−1^ BAP (PM1, 71 ± 10.2%) and 5.0 mg L^−1^ BAP (PM3, 73.3 ± 11.6%). The quality of multiple buds was also better on the lower concentration of BAP. Explants cultured on MS medium with BAP and without activated charcoal (P4–P6) showed a response of bud induction (Table 3). The buds were of poor quality, and all turned brown after sub-culturing on the same medium without activated charcoal. This result indicated that supplementation of activated charcoal in the medium had an important role in bud induction, quality, and further development. It was also observed that induction of multiple buds depended on the cutting of explants.

**Table 3.** Effect of various concentrations of BAP on multiple bud induction and shoot regeneration of *Ensete ventricosum* (Welw.) Cheesman (enset) using corm discs

	Multiple bud induction medium	BAP (mg L^−1^)	Activated charcoal (2 g L^−1^)	Efficiency of bud induction at 4 wk (%)^za^	Shoot regeneration medium	BAP (mg L^−1^)	Activated charcoal (2 g L^−1^)	Number of shoots initiated per corm disc^a^	Number of complete plants per corm disc^a^	Multiplication rate^ya^
1	PM2	1.0	Yes	81.7 ± 14.1	PM2	1.0	Yes	29.6 ± 1.6	12.0 ± 0.9	36.1 ± 2.8
2	PM1	2.5	Yes	71.0 ± 10.2	PM1	2.5	Yes	26.3 ± 2.9	4.6 ± 0.5	13.7 ± 1.5
3	PM3	5.0	Yes	73.3 ± 11.6	PM3	5.0	Yes	33.5 ± 8.9	4.3 ± 0.9	13.0 ± 2.8
4	PM4	1.0	No	68.7 ± 10.3	PM4	1.0	No	0 ± 0	0 ± 0	0 ± 0
					PM2	1.0	Yes	28.4 ± 3.9	0.9 ± 0.1	2.8 ± 0.4
5	PM5	2.5	No	75.3 ± 4.0	PM5	2.5	No	0 ± 0	0 ± 0	0 ± 0
					PM1	2.5	Yes	28.7 ± 4.1	0.4 ± 0.1	1.1 ± 0.3
6	PM6	5.0	No	64.0 ± 3.5	PM6	5.0	No	0 ± 0	0 ± 0	0 ± 0
					PM3	5.0	Yes	47.4 ± 4.3	0.7 ± 0.1	2.1 ± 0.2
7	MBM1	0.0	Yes	55.3 ± 4	MBM1	0.0	Yes	42.3 ± 2.9	11.6 ± 0.36	34.9 ± 1.1
8	MBM2	0.0	No	50.7 ± 4	MBM2	0.0	No	30.6 ± 0.7	1.8 ± 0.2	5.3 ± 0.6

^a^Values are mean ± Std.

^z^Efficiency of bud induction (%) = (number of explants producing multiple buds / total number of explants cultured) × 100.

^y^Multiplication rate = total number of plantlets regenerated from three corm discs isolated from one *in vitro* mother plantlet.

The buds obtained from a medium containing 1.0, 2.5, or 5.0 mg L^−1^ BAP supplemented with activated charcoal produced shoots when transferred to the same fresh medium. About 26–33 shoots were initiated from each corm disc. However, only a few developed into complete plantlets (Table 3). About 12 complete plantlets were obtained from each corm disc on a medium containing 1 mg L^−1^ BAP and supplemented with activated charcoal, whereas only four were obtained when a higher concentration of BAP was used.

No shoots were initiated from the multiple buds induced on a medium without activated charcoal (P4–P6, Table 3). All of these buds turned brown, and further development into shoots was arrested. However, when these buds were transferred to the same medium with activated charcoal (P1–P3), shoot initiation was observed. Even though shoots were developed, only one to three shoots developed into complete plantlets (Table 3). The present results corroborate the work of Negash *et al.* (2000) and Birmeta and Welander (2004) which showed that the addition of activated charcoal in the culture medium was very effective in controlling the blackening of explants without an adverse effect on the regeneration of enset.

In this study, multiple buds were obtained on various types of media. However, the buds obtained on the CIM1 medium showed the highest regeneration efficiency, with about 36 complete plantlets per corm disc. As three corm discs were isolated from each mother plantlet, a total of about 104–108 plantlets were produced from each mother plantlet in 4 mo **(**Table 2). A higher multiplication rate was obtained compared with previous reports on enset. Negash *et al.* (2000) reported production of an average of 31 plantlets per corm in 16 wk. However, Birmeta and Welander (2004) reported 75 shoots from corm tissues in 14 wk. Konobo (2014) reported production of 2–15 shoots for different enset cultivars using shoot tips as explants.

Regenerated shoots were transferred to proliferation medium with activated charcoal (PM2) for multiplication and development of complete plants. All of the shoots developed into well-rooted plantlets on PM2 medium in 4 wk (Fig. 4 *D*). Fully developed rooted plantlets were weaned and hardened for 4 wk (Fig. 4 *e*) then transferred in week 5 to large pots for acclimatization (Fig. 4 *f*). Plants regenerated through corm discs were normal, and no phenotypical abnormalities were observed in the potted plants.

The final protocol for regeneration was validated by being repeated by various researchers in the group. The schematic flow diagram for regeneration of complete plants using corm discs is presented in Fig. 5. Currently, this protocol is being validated with other farmer-preferred cultivars of enset.

**Figure 5.**
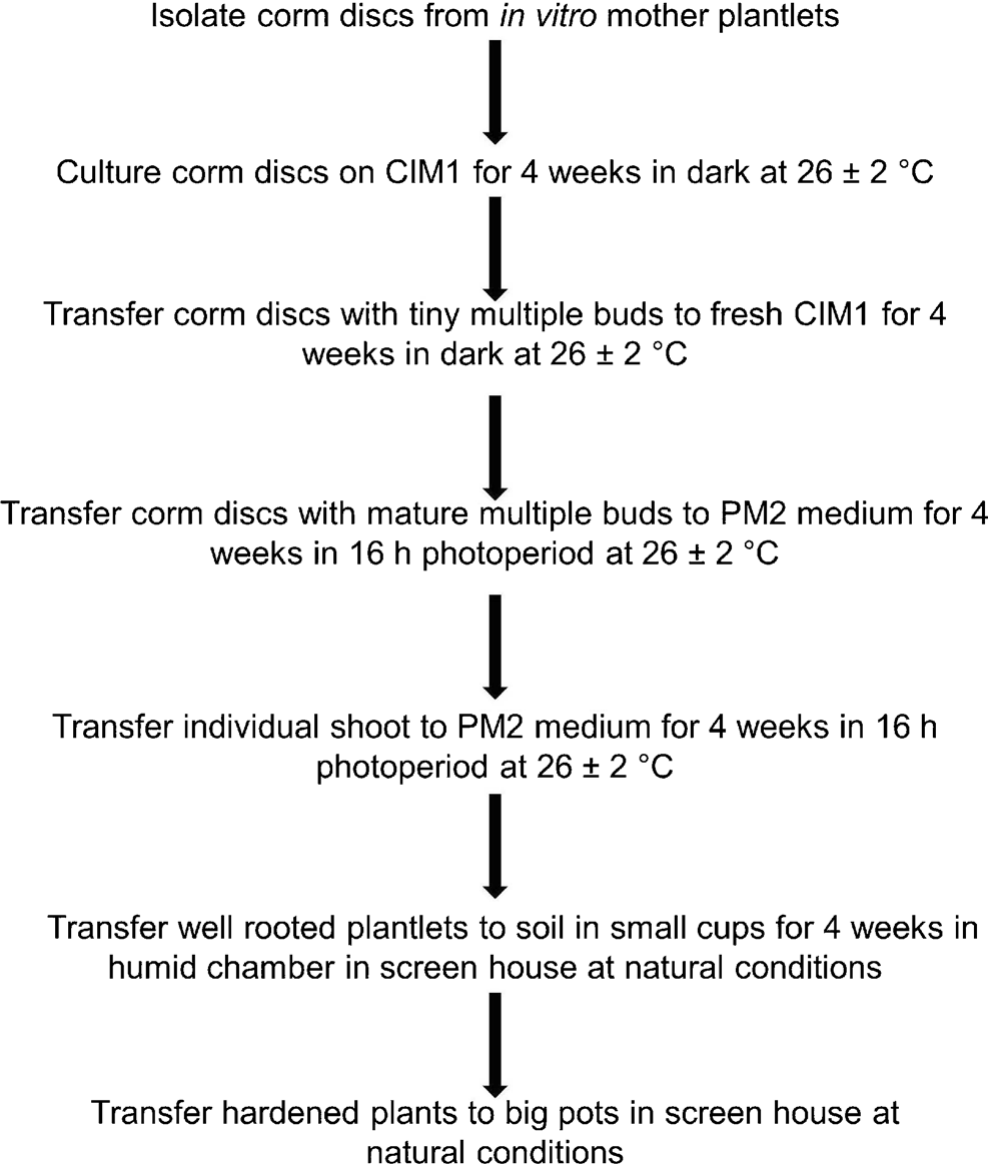
Schematic flow diagram showing various steps of regeneration of complete plants of *Ensete ventricosum* (Welw.) Cheesman (enset) using corm discs isolated from *in vitro* mother plantlets.
